# Prognostic Significance of Organ Dysfunction in Cats With Polytrauma

**DOI:** 10.3389/fvets.2019.00189

**Published:** 2019-06-21

**Authors:** Elsa Murgia, Roberta Troia, Cecilia Bulgarelli, Marco Pelizzola, Armando Foglia, Francesco Dondi, Massimo Giunti

**Affiliations:** Department of Veterinary Medical Sciences, Alma Mater Studiorum, University of Bologna, Bologna, Italy

**Keywords:** polytrauma, multiple organ dysfunction syndrome, shock, severity scoring systems, feline, prognosis

## Abstract

Polytrauma is a common emergency condition in small animals and is frequently associated with higher morbidity and mortality rates compared to minor trauma. Multiple Organ Dysfunction Syndrome (MODS) is a major complication of extensive traumatic injury, carrying a high risk of death despite intensive care treatment. Little is known about the prevalence and the prognostic impact of MODS in feline polytrauma. The current study aimed to prospectively evaluate the occurrence and the prognostic significance of organ dysfunction at admission in a population of polytraumatized cats. Cats with polytrauma requiring intensive care unit hospitalization were included and categorized according to outcome (survivors/non-survivors). Clinical and clinicopathological data, including scores of disease severity [Animal Trauma Triage Score (ATTS), APPLE_fast_, and APPLE_full_], selected organ dysfunction and presence of MODS were evaluated upon admission, and analyzed with respect to mortality. Non-parametric statistics was performed and *P* < 0.05 was considered significant. Thirty-eight cats met the inclusion criteria: 8/38 (21%) had penetrating trauma, while 30/38 (79%) had blunt trauma. The overall in-hospital mortality was 37% (14/38). Cats with evidence of MODS upon admission had significantly higher frequency of death compared to cats without MODS (9/14 vs. 2/24 *P* = 0.0004). Hemostatic dysfunction, respiratory dysfunction, and MODS upon admission were significantly associated with mortality in the univariate logistic regression analysis (*P* = 0.005, *P* = 0.001, *P* = 0.001, respectively). The values of APPLE_fast_, APPLE_full_, and ATTS were independently associated with a higher risk of death and positively correlated with the number of dysfunctional organs (*P* = 0.025, *P* = 0.004, *P* = 0.003, *r* = 0.57, *P* = 0.0002; *r* = 0.59, *P* = 0.0001; *r* = 0.55, *P* = 0.0003, respectively). Multiple Organ Dysfunction Syndrome is a common complication of feline polytrauma and its development is associated with increased disease severity and worse outcomes. The presence of hemostatic dysfunction and respiratory dysfunction upon admission is associated with a higher risk of death. The ATTS and the APPLE scores are useful prognostic tools for the assessment of cats with polytrauma.

## Introduction

Polytrauma is a common presenting condition in the human intensive care unit (ICU), with variable morbidity and mortality rates (1). Patients surviving primary traumatic injury can develop systemic life-threatening complications such as sepsis, hemorrhage, and multiorgan dysfunction syndrome (MODS). Among these, MODS represents a frequent cause of early and late death in up to the 40% of human ICU patients after severe trauma, and its occurrence is related to the severity and the type of the trauma (2–4).

Trauma is one of the most prevalent disorders presented both to referral and primary veterinary practices (5). Overall mortality rates in feline trauma patients range from 12 to 22% (5–11). Polytraumatic injuries have been reported to occur in 25–64% of feline trauma patients (5, 10) and were associated with a higher risk of death compared to minor trauma (17.2 vs. 2.9%) (10). There is little information in the veterinary literature regarding MODS following trauma. In two retrospective studies including dogs with penetrating and blunt trauma, respectively, the presence of MODS and selected organ dysfunction including respiratory, hemostatic, and cardiovascular dysfunctions were significantly associated with a worse prognosis (12–14). To our knowledge, the impact of organ dysfunction has never been investigated in cats with polytrauma. The purpose of this study was, therefore, to prospectively evaluate the prevalence and the prognostic significance of organ dysfunction at admission in a population of polytraumatized cats. We hypothesized that MODS is a frequent complication of feline polytrauma and that its development is associated with increased disease severity and worse outcome.

## Materials and Methods

This was a prospective, observational study investigating polytrauma in a feline population admitted to the emergency room of a Veterinary University Hospital (VUH) between September 2015 and September 2017. The study was approved by the local Institutional Animal Care and Use Committee.

### Animals

Cats were eligible for inclusion in the study if they had a history and clinical signs consistent with polytrauma that had occurred in the previous 24 h. Polytrauma was defined as the simultaneous presence of clinically significant injuries to multiple body regions or cavity, compromising the patient's physiology (5, 10) and requiring ICU admission. Patients were excluded if treatments had been provided prior to presentation.

### Data Collection

Attending ICU clinicians were responsible for the clinical management of the patients included in the study. At the time of presentation at our VUH, the following data were recorded: signalment and history, including: time from trauma to arrival at the emergency room, type and location of injuries, type of trauma (blunt or penetrating trauma), and clinical findings including rectal temperature, heart rate, respiratory rate, and mental status. Abdominal and thoracic-focused assessment with sonography for trauma (AFAST and TFAST, respectively) were performed using point-of-care ultrasound (Z5Vet with micro-convex 6.5 MHz, Mindray, Shenzhen, China), as previously described (15). Non-invasive blood pressure measurement was performed using an oscillometric method on the thoracic limb (petMAT^TM^ graphic, Ramsey Medical, Sydney, Australia) and double-checked using a Doppler technique (Minidop ES-100 VX, Hadeco, Kawasaki, Japan). The SpO_2_ measurement was performed with a pulse CO-oximeter (Masimo RAD-57, California, USA). Blood was collected by venipuncture with a vacuum system, according to standard operating procedures, and the following analyses were performed: venous blood gas analysis, including electrolyte and lactate concentrations (ABL 800 FLEX, Radiometer Medical ApS, Copenhagen, Denmark), complete blood count (ADVIA 2120, Siemens Healthcare Diagnostics, Erlangen, Germany) and microscopical evaluation of the blood smear, chemistry profile including measurement of serum creatinine and serum bilirubin (AU 480, Beckman Coulter-Olympus, Brea, California, USA), and coagulation profile including prothrombin time (PT) and activated partial thromboplastin time (aPTT) (BFT II, Siemens, Munich, Germany). Blood collection was performed prior to fluid resuscitation and drug administration whenever possible. In selected cases of severe hemodynamic or respiratory impairment, sampling was performed during or soon after the first stabilization procedures, within 1 h from arrival. Endpoints of resuscitation included the restoration of clinical perfusion parameters (e.g., heart rate, peripheral pulse quality, and rectal temperature), blood pressure, and urine output. Additionally, blood lactate concentration was checked for evaluation of resuscitative efforts. The animal trauma triage score (ATTS) and the feline acute patient physiologic and laboratory evaluation (APPLE) scores, APPLE_fast_ and APPLE_full_, respectively, were calculated, as previously described (16, 17). Additional data recorded included radiological findings, need for fluid resuscitation, administration of blood products, and treatments administered during hospital stay. Cats were classified according to their outcome as survivors (alive to discharge) and non-survivors (died despite medical treatment or humanely euthanized because of impending cardiopulmonary arrest). Cats euthanized for financial or other than ethical reasons were excluded from the study.

### Organ Dysfunction

The criteria to define and classify dysfunction of different organs were adapted from the available canine literature (18–20). Cut-off values for specific variables were based on the upper limit of the reference intervals (RI) of our clinical pathology laboratory: total bilirubin RI 0–5.98 μmol/L; PT RI 9–15 s; aPTT RI 9–20 s. The following organ dysfunctions were considered: (a) cardiovascular dysfunction: hypotension (systolic blood pressure <90 mmHg) in volume-resuscitated patients requiring support with inotropes or vasopressors; (b) hemostatic dysfunction: PT > 15 s and/or aPTT > 20 s and/or platelet count <100,000/mm^3^ in the absence of clumps on evaluation of the blood smear; (c) liver dysfunction: serum bilirubin >5.98 μmol/L in absence of hemolysis or biliary obstruction/rupture (based on a complete abdominal ultrasound); d) renal dysfunction: presence of acute kidney injury (AKI), defined as serum creatinine (sCr) >141.44 μmol/L, and/or increase in sCr of ≥26.52 μmol/L from baseline and/or oliguria (urine output <1 mL/kg/h over 6 h) (20); (e) respiratory dysfunction: signs of respiratory distress associated with an SpO2 <95% on room air, need for oxygen supplementation and/or mechanical ventilation support; (f) MODS: presence of at least two dysfunctional organs, simultaneously.

Clinical and laboratory variables, including the presence of selected organ dysfunction, occurrence of MODS, the total number of dysfunctional organs and the clinical scores (ATTS, APPLE_fast_, and APPLE_full_) were assessed for outcome prediction at the time of presentation.

### Statistical Analysis

Data distribution was assessed graphically and using the D'Agostino-Pearson test. Descriptive statistics were evaluated as appropriate. Since most variables were not normally distributed, data were expressed as median and range (min-max), and non-parametric tests were used for comparisons. The Fisher's exact test and the Mann–Whitney *U*-test were used to compare categorical and continuous variables, respectively, among groups. Receiver operating characteristic (ROC) curve analysis was used to find optimal cut-off values for variables predicting prognosis and to calculate the area under the ROC curve (AUC). Univariate linear regression analysis was used to evaluate significant variables in respect to outcome prediction (stepwise approach). Correlations between continuous variables were determined using the Spearman's rank correlation coefficient; a value of *P* < 0.05 was considered significant. Statistical analysis was performed using a statistical software package [MedCalc Statistical Software version 15.8 (MedCalc Software bvba, Ostend, Belgium; https://www.medcalc.org; 2015)].

## Results

A total of 113 traumatized cats were admitted to the emergency room during the study period. Seventy-five subjects were excluded from this study for the following reasons: previous treatments by the referring veterinarians (2/113, 2%), presentation >24 h after the traumatic incident (2/113, 2%), incomplete data collection (2/113, 2%), euthanasia for financial reasons (2/113, 2%), presence of minor injuries that did not require admission to the ICU (67/113, 59%). Ultimately, 38 cats met the criteria for inclusion in this study. The median age was 3 years (0.58–18). There were 19/38 (50%) males (8 neutered and 11 intact) and 19/38 (50%) females (11 neutered and 8 intact). The majority of cats were mixed breed 37/38 (97%) and only one was purebred 1/38 (3%) (Maine Coon). The median bodyweight was 4 kg (2.5–8.5) and median time from trauma to presentation to the hospital was 4 h (0.5–24). Blunt trauma was identified in 30/38 (79%) cats, while penetrating trauma was diagnosed in the remaining 8/38 (21%) animals. No association between type of trauma and median time to hospital presentation was found. Of the 30/38 cases with blunt trauma, causes included road traffic accidents (19/30, 63%), falls from heights (7/30, 23%), crush injuries (2/30, 7%), and unknown trauma (2/30, 7%). Concerning the 8/38 cases with penetrating trauma, 6/8 (75%) had bite wounds, 1/8 (12.5%) had stab wounds, and 1/8 (12.5%) had gunshot trauma. Selected clinical and clinicopathological variables recorded upon hospital admission in the study population and in cats classified as survivors and non-survivors are reported in Table 1. Trauma involved multiple areas, including extremities and pelvis (23/38, 61%), thorax (17/38, 45%), head and face (12/38, 32% and 7/38, 18%, respectively), skin (9/38, 24%) abdomen (8/38, 21%), and spine (4/38, 11%), with some cats counted in multiple categories. Effusions were detected in 9/38 (24%) cats by TFAST and in 7/38 (18%) cats by AFAST. None of the cats with a positive TFAST required thoracocentesis based on their breathing pattern and/or pulse oximetry. Sampling and analysis of the abdominal effusions was possible in four cases, revealing uroabdomen in one cat and hemoabdomen in the remaining three cats. Pneumothorax requiring thoracentesis was identified by TFAST in 2/38 (5%) cats. Fluid resuscitation for hypovolemic shock was required in 24/38 (63%) cats upon admission. Mean volume of fluid given during fluid resuscitation was 63.5 ± 33.9 ml per cat. Ringer's lactate was the most frequently used solution (22/24, 92%) with a mean volume of 65 ± 32 ml, followed by 7.5% hypertonic saline (5/24, 21%) with a median amount of 20 ml (range, 10–22), and tetrastarch (8.4 ml of 6% hydroxyethyl starch 130/0.42, Amidolite®) (1/24, 4%). Twenty-three out of 24 cats (96%) responded within 1 h of initiation of fluid resuscitation, while one cat with bite trauma developed cardiovascular dysfunction requiring vasopressor support with norepinephrine for ~14 h. Packed red blood cells and fresh frozen plasma were administered during the hospital stay in 2/38 and 1/38 cats, respectively, to correct anemia and to replete coagulation factors in a bleeding cat.

**Table 1:** Descriptive statistics for selected variables measured in cats with polytrauma at the hospital presentation (*n* = 38); cats were classified as survivors (*n* = 24) and non-survivors (*n* = 14).

**Variable**	**Study population (*n* = 38)**	**Survivors (*n* = 24)**	**Non-survivors (*n* = 14)**	**R.I**.	***P*-value**
Rectal body temperature (°C)	36.7 (32–39.6)	37.6 (33.1–39.6)	34.5 (32–38.6)	37.8–39.5	0.02
Heart rate (beats/min.)	177 (96–280)	180 (96–280)	150 (120–240)	140–200	0.13
Respiratory rate (breaths/min.)	48 (16–130)	46 (16–130)	54 (20–120)	20–40	0.46
Systolic blood pressure (mmHg)	126 (60–265)	130 (60–179)	70 (60–265)	120–170	0.02
ATTS	5 (1–13)	4 (1–8)	8 (4–13)	NA	0.0001
APPLE_fast_ score	34 (19–50)	29.5 (19–46)	39.5 (21–50)	NA	0.03
APPLE_full_ score	50 (35–70)	48.5 (35–64)	54.5 (39–70)	NA	0.009
**Hematology**					
HCT (%)	35.9 (20.4–42.9)	35.9 (20.4–42.2)	36.4 (25.3–42.9)	32–48	0.69
Platelets (cells ×10^3^/mm^3^)	248 (14.2–637)	242 (51–515)	251 (14.2–637)	50–500	0.89
**Chemistry**					
Creatinine (μmol/L)	24.8 (10.1–65.7)	22.2 (14.9-65.7)	30.1 (10.1-61.7)	13.7-30.8	0.21
Total bilirubin (μmol/L)	2.39 (0.17–25.82)	2.39 (0.17–8.2)	2.39 (1.54–25.82)	0–5.98	0.41
Lactate (mmol/L)	2.75 (0.8–14.7)	2.4 (0.8–14.7)	3.9 (1.9–11.6)	0.5–2	0.01
Glucose (mmol/L)	12.0 (4.8–22.7)	12.2 (4.8–22.7)	10.4 (5.6–22.1)	4–9	0.36
**Coagulation profile**					
PT (sec)	11.0 (8.8–31.0)	10.8 (9.2–15.2)	12.1 (8.8–31.0)	9–15	0.04
aPTT (sec)	14.2 (4.9–97.0)	13.6 (4.9–83.0)	20.6 (10.7–97.0)	9–20	0.046
**Blood gas analysis (venous)**					
pH	7.24 (6.67–7.47)	7.26 (7.17–7.47)	7.21 (6.67–7.29)	7.31–7.46	0.017
pCO_2_ (kPa)	5.5 (3.2–9.6)	5.3 (3.2–7.9)	5.8 (3.8–9.6)	4.4–6.0	0.38
HC${\text{O}}_{3}^{-}$ (mmol/L)	17.2 (4.5–23.0)	17.6 (10.2–23.0)	16.6 (4.5–22.7)	18.0–22.0	0.46
Base Excess	−8.7 (−29.0 to −0.9)	−7.6 (−16.0 to −0.9)	−9.2 (−29.0 to −3.7)	−2.0 to +2.0	0.15
**Additional data**					
Days of ICU	2.5 (0–15)	3 (1–8)	1 (0–15)	NA	0.02
Number of ODs	1 (0–4)	0 (0–3)	2 (0–4)	NA	0.0002

*Values for each analyte are presented as median and (range). ATTS, Animal Trauma Triage Score; APPLE, feline Acute Patient Physiologic and Laboratory Evaluation; HCT, hematocrit value; ICU, intensive care unit; ODs, organ dysfunctions; PT, prothrombin time; aPTT, thromboplastin activity time; NA, not applicable; R.I., reference interval. P-value <0.05 indicates a significant difference between survivors and non-survivors*.

There were 14 non-survivors; two cats were euthanized due to a perceived grave prognosis, while 12 cats experienced cardiopulmonary arrest and spontaneous death. Nine out of 14 cats (64%) died within 24 h of admission. No significant association between time elapsed between trauma and admission to the VUH or type of trauma (blunt vs. penetrating) and mortality was identified.

A significantly higher frequency of need for fluid resuscitation, judged by the attending clinician, was reported in non-survivors compared to survivors (12/14, 86% vs. 12/24, 50%, *P* = 0.04); however, the median volume of resuscitation fluids was not significantly different between the two groups (69.5 mL, range 20–136 vs. 51 mL, range 20–133, respectively, *P* = 0.2).

The ATTS, the APPLE_fast_, and the APPLE_full_ scores at presentation were independently associated with a higher risk of death in the univariate logistic regression analysis (Table 2). The ROC curve analysis revealed that values of ATTS higher than 5 (sensitivity 78.6%, specificity 79.2%; AUC = 0.87), values of APPLE_fast_ Score > 37 (sensitivity 71.4%, specificity 79.2%; AUC = 0.71), and values of APPLE_full_ > 53 (sensitivity 64.3%, specificity 83.3%; AUC = 0.75) were able to predict mortality upon admission.

**Table 2:** Univariate binary logistic regression analysis results of variables associated with outcome (survivors/non-survivors) in 38 cats with polytrauma.

**Variable**	**RC**	**SE**	**Odds ratio [95% CI]**	***P*-value**
MODS	2.98	0.92	19.8 (3.227–121.47)	0.001
Number of dysfunctional organs	1.54	0.5	4.68 (1.736–12.663)	0.002
Hemostatic dysfunction	2.20	0.78	9.0 (1.944–41.655)	0.005
Respiratory dysfunction	3.72	1.16	41.4 (4.23–405.233)	0.001
ATTS	0.8	0.27	2.23 (1.309–3.802)	0.003
APPLE_fast_ score	0.1	0.05	1.10 (1.006–1.218)	0.04
APPLE_full_ score	0.14	0.06	1.16 (1.03–1.299)	0.01

*CI, confidence interval; RC, regression coefficient; SE, standard error; ATTS, Animal Trauma Triage Score; APPLE, feline Acute Patient Physiologic and Laboratory Evaluation; MODS, Multi Organ Dysfunction Syndrome*.

### Organ Dysfunction and Outcome

The frequencies of organ dysfunction reported in the study population at presentation were the following: hemostatic (13/38, 34%), renal (12/38, 32%), respiratory (10/38, 26%), liver (5/38, 13%), and cardiovascular (1/38, 3%). Twenty-two out of 38 (58%) cats had at least one organ dysfunction. In our study population, evidence of thoracic trauma was documented by radiological findings in only 6/10 (60%) cats with respiratory dysfunction and was associated with the presence of pulmonary contusions (*n* = 6), rib fractures (*n* = 3), pneumothorax (*n* = 2), and Veterinary Acute Lung Injury (VALI) (*n* = 1), based on previously defined criteria (21). Four out of 10 (40%) cats with respiratory dysfunction were presented for head trauma. Arterial blood gas analysis was available only for the cat with VALI, showing hypoxemia (PO_2_ = 8.39 kPa) and hypercapnia (PCO_2_ = 9.59 kPa) on room air. Mean venous PCO_2_ in the rest of the patients (*n* = 37) was 5.47 ± 1.1 kPa. Respiratory and hemostatic dysfunction were both the most represented organ dysfunction in non-survivors (9/14, 64%). However, the presence of the latter was not correlated with body cavity hemorrhage, positive AFAST or TFAST findings, the need for blood product administration or antifibrinolytic drugs (data not shown). According to the criteria for the definition of acute traumatic coagulopathy (ATC), previously adopted by Holowaychuk et al. (22), it was possible to diagnose this syndrome in 7/38 cats of our population. Conversely, no cat in our study fulfilled the criteria of ATC proposed by Gottlieb et al. (9). All cats with renal dysfunction (12/38; 32%) were presented with an increased serum creatinine concentration. Additionally, cats with renal dysfunction compared to those without renal dysfunction had significantly lower systolic blood pressure (70 mmHg, 70–149 vs. 130 mmHg, 60–265; *P* = 0.01) and base excess (−12.5, range −20 to −7.7 vs. −7.2, range −29 to −0.9; *P* = 0.0007) and significantly higher blood lactate concentrations (6.1, 1.1–14.7 vs. 2.4, 0.8–11.6; *P* = 0.003), respectively. Moreover, 10/12 (83%) cats with renal dysfunction needed fluid resuscitation at presentation.

The presence of MODS was documented in 11/38 (29%) cats upon admission, and the numbers of dysfunctional organs were two in 7/11 cats, three in 3/11 cats, and four in 1/11 cat. Organ dysfunction documented in cats with MODS were the following: respiratory (10/11), hemostatic (8/11), renal (7/11), hepatic (1/11), and cardiovascular (1/11). The number of dysfunctional organs upon admission was significantly higher in non-survivors compared to survivors (*P* = 0.0002) (Table 1). Patients with evidence of MODS upon admission had a significantly higher frequency of death compared to patients without MODS (9/14, 64% vs. 2/24, 8%, *P* = 0.0004). According to the results of the univariate logistic regression, there were positive associations between odds of mortality and the presence of hemostatic dysfunction, respiratory dysfunction and MODS (Table 2). Positive correlations between organ dysfunction and the scores of disease severity are reported in Table 3. Among survivors, the presence of hemostatic dysfunction, the number of dysfunctional organs, the APPLE_fast_ score and the need of fluid resuscitation at presentation were positively correlated with a longer ICU stay (*r* = 0.51; *P* = 0.01; *r* = 0.61, *P* = 0.001; *r* = 0.44, *P* = 0.03; *r* = 0.52, *P* = 0.009, respectively).

**Table 3:** Significant correlations documented between selected organ dysfunction and the scores of disease severity upon admission in 38 cats with polytrauma.

**Variable**	**ATTS score**	**APPLE_**fast**_ score**	**APPLE_**full**_ score**
Hemostatic dysfunction	*r* = 0.48	*r* = 0.43	*r* = 0.42
	*P* = 0.002	*P* = 0.006	*P* = 0.007
Respiratory dysfunction	*r* = 0.63	NS	*r* = 0.37
	*P* < 0.01		*P* = 0.02
Renal dysfunction	NS	*r* = 0.39	NS
		*P* = 0.01	
Hepatic dysfunction	NS	*r* = 0.38	*r* = 0.33
		*P* = 0.01	*P* = 0.03
Cardiovascular dysfunction	NS	NS	NS
MODS	*r* = 0.56	*r* = 0.38	*r* = 0.38
	*P* < 0.01	*P* = 0.01	*P* = 0.01
Number of dysfunctional organs	*r* = 0.55	*r* = 0.57	*r* = 0.59
	*P* < 0.001	*P* < 0.001	*P* < 0.001

*ATTS, Animal Trauma Triage Score; APPLE, feline Acute Patient Physiologic and Laboratory Evaluation; MODS, Multi Organ Dysfunction Syndrome; NS, non-significant*.

## Discussion

The present study describes a population of polytraumatized cats hospitalized in a veterinary ICU, focusing on the presence of organ dysfunction at admission and its impact on mortality.

This study documented a greater proportion of blunt trauma (79%) compared to penetrating trauma (21%). Among blunt injuries, road traffic accidents were the most common, according to the previous veterinary literature (7, 8, 10, 23). Cats included in our study population frequently had clinical signs consistent with shock and shock-associated metabolic derangements including metabolic acidosis, hyperlactatemia, hyperglycemia, and increased liver enzyme concentrations (data not shown), as previously reported (10, 24, 25).

The mortality rate for polytraumatized cats in the present study was higher than the one reported in a previous study (10); however, differences in the study populations, types of injuries, and euthanasia should be taken into account when comparing the results of the two studies. The median time between trauma and hospital admission was 4 h, and no significant association between mortality and duration of ICU stay, respectively, was noted.

Our study confirmed that in cats with polytrauma, a more severe degree of shock upon arrival (documented by lower blood pressure and body temperature), higher blood lactate concentration, increased need for fluid resuscitation, and higher clinical scores of disease severity, occurred more frequently in non-survivors, as previously reported (10). Interestingly, the administered volume of resuscitation fluids was not different between survivors and non-survivors. Nevertheless, that finding does not rule out a more severe tissue hypoperfusion in the non-survivors. Moreover, no significant complications related to fluid overload (e.g., pleural effusion or pulmonary edema) were found in our population, but the impact of the water balance on the final outcome was not evaluated.

Scoring systems are increasingly applied in intensive care (26). Regarding veterinary trauma patients, ATTS was able to predict the short-term outcome and the need for intensive care in a previous study of dogs and cats suffering from gunshot injuries (27). Studies focusing on the utility of the APPLE scores in traumatized cats are lacking. In the present study, the ATTS, the APPLE_fast_, and the APPLE_full_ scores were calculated at the time of admission for prognostic purposes. According to our results, greater values of all of the above scores were documented in non-survivors. According to the results of the ROC curve analysis, ATTS had a good accuracy to correctly predict the outcome, while the accuracy for the APPLE scores was only fair. Furthermore, all investigated scores correlated with the presence of MODS or with selected organ dysfunction. Such findings highlight their role in the identification of the more severely affected patients and justify their use as a component of the overall clinical assessment of cats with polytrauma.

Multiple organ dysfunction was fairly common in the current study population, as 11 out of 38 (29%) cats had MODS at the time of inclusion. In addition, the presence of MODS was associated with an increased risk of mortality. These results are novel in the course of feline polytrauma and are similar to the data reported in people and dogs (12, 13, 28, 29).

Acute traumatic coagulopathy (ATC) is a poorly defined syndrome associated with trauma and shock, that usually contributes to post-traumatic hemorrhage and fatal complications in humans (30, 31). A paucity of studies documents ATC in polytraumatized animals, with variable associations between its occurrence, the presence of clinical bleeding, the necessity of blood products, and the severity of trauma (9, 22). A previous study found that ATC is rare in minimally injured cats following blunt trauma, and higher disease severity scores (ATTS) did not appear to predict coagulopathy (9). The criteria used in this study to define hemostatic dysfunction may have underestimated the presence of ATC. Nevertheless, according to our results, the presence of hemostatic dysfunction was associated with a worse prognosis and should not be underestimated, even in the absence of clinical bleeding. The criteria adopted to define hemostatic dysfunction in our study have intrinsic limits for being able to predict hemorrhage or risk of hemorrhage, since the variables (e.g., PT and aPTT) were not designed for that purpose (32). While there is evidence that thromboelastography and rotational thromboelastometry might better predict bleeding than standard tests, a recommendation on the definition of hypocoagulability, based on the former ones, is lacking thus far (32). Therefore, future studies are needed to better evaluate and characterize hemostatic dysfunction in cats with polytrauma.

Renal dysfunction was the second most common organ dysfunction documented upon admission in our population, but did not show any prognostic relevance at that time. All cats with renal dysfunction were azotemic and showed more severe signs of hypoperfusion, documented by lower values of systolic blood pressure and base excess, and higher blood lactate concentration compared to cats without renal dysfunction. Moreover, fluid resuscitation upon arrival was required in most of the cats with renal dysfunction. However, the early death of the majority of these cases did not allow extensive characterization of kidney injury features (e.g., volume responsiveness vs. intrinsic damage) in our study population. No epidemiological data concerning traumatic AKI are reported in cats, and the criteria to define renal dysfunction differ among veterinary studies (13, 33). Although renal dysfunction did not carry any prognostic impact in the current study, future studies are needed to better characterize AKI in polytraumatized cats.

In this study, the presence of respiratory dysfunction was significantly associated with a higher risk of death. According to human and canine literature, respiratory impairment is a common complication in the polytraumatized patient, with thoracic trauma representing a frequent cause of death in this setting (13, 34, 35). The evaluation of respiratory dysfunction, however, was contingent upon a certain degree of subjectivity; the presence of hypoxemia was evaluated by pulse oximetry and just in one case confirmed by arterial blood gas measurement, while moderate/severe hypercapnia was observed in a minority of the patients. Thus, this limitation should be taken in account when interpreting our results.

There are some limitations to consider when interpreting our results. The criteria used to define organ dysfunction, although based on and adapted from the available canine literature, could be improved and need to be validated in a wider population of feline patients.

## Conclusion

The current study highlights the prognostic role of selected organ dysfunction and MODS in a population of polytraumatized cats. Specifically, renal, hemostatic, and respiratory dysfunction were common in cats with polytrauma at the time of presentation. Additionally, hemostatic dysfunction, respiratory dysfunction, presence of MODS, and the overall number of dysfunctional organs, upon admission were associated with a higher mortality. The ATTS, the APPLE_fast_, and the APPLE_full_ scores calculated at presentation correlated positively with MODS, were independently associated with a higher risk of death, and should be evaluated to maximize intensive care in this setting. Further larger studies aiming at characterizing MODS in cats with polytrauma are warranted. Moreover, to improve prognostic performance of the criteria of organ dysfunction, they should be graded according to the severity of the dysfunction and not only as being present or absent.

## Ethics Statement

The project was approved by the Animal Welfare Committee (COBA) of the *Alma Mater Studiorum*—University of Bologna (Bologna DL 26/2014, Project 847).

## Author Contributions

EM, MG, and RT designed the study, analyzed data, co-wrote, and edited the manuscript. CB, MP, and AF assisted with study design, collected, and analyzed data. FD analyzed data and edited the manuscript. All authors contributed to read and approved the final manuscript.

### Conflict of Interest Statement

The authors declare that the research was conducted in the absence of any commercial or financial relationships that could be construed as a potential conflict of interest.

## Abbreviations

AKI: Acute Kidney Injury
APPLE: Acute Patient Physiologic and Laboratory Evaluation
ATC: Acute Traumatic Coagulopathy
ATTS: Animal Trauma Triage Score
MODS: Multiple Organ Dysfunction Syndrome
SIRS: Systemic Inflammatory Response Syndrome.
